# Sirtuins and Sepsis: Cross Talk between Redox and Epigenetic Pathways

**DOI:** 10.3390/antiox11010003

**Published:** 2021-12-21

**Authors:** Anugraha Gandhirajan, Sanjoy Roychowdhury, Vidula Vachharajani

**Affiliations:** 1 Department of Inflammation and Immunity, Lerner Research Institute, Cleveland Clinic, Cleveland, OH 44195, USA; gandhia3@ccf.org (A.G.); roychos@ccf.org (S.R.); 2 Department of Critical Care Medicine, Respiratory Institute, Cleveland Clinic, Cleveland, OH 44195, USA

**Keywords:** sepsis, immune response, oxidative stress, mitochondrial dysfunction, epigenetic programming, sirtuins, hyper-inflammation, hypo-inflammation

## Abstract

Sepsis and septic shock are the leading causes of death among hospitalized patients in the US. The immune response in sepsis transitions from a pro-inflammatory and pro-oxidant hyper-inflammation to an anti-inflammatory and cytoprotective hypo-inflammatory phase. While 1/3rd sepsis-related deaths occur during hyper-, a vast majority of sepsis-mortality occurs during the hypo-inflammation. Hyper-inflammation is cytotoxic for the immune cells and cannot be sustained. As a compensatory mechanism, the immune cells transition from cytotoxic hyper-inflammation to a cytoprotective hypo-inflammation with anti-inflammatory/immunosuppressive phase. However, the hypo-inflammation is associated with an inability to clear invading pathogens, leaving the host susceptible to secondary infections. Thus, the maladaptive immune response leads to a marked departure from homeostasis during sepsis-phases. The transition from hyper- to hypo-inflammation occurs via epigenetic programming. Sirtuins, a highly conserved family of histone deacetylators and guardians of homeostasis, are integral to the epigenetic programming in sepsis. Through their anti-inflammatory and anti-oxidant properties, the sirtuins modulate the immune response in sepsis. We review the role of sirtuins in orchestrating the interplay between the oxidative stress and epigenetic programming during sepsis.

## 1. Introduction

Sepsis is the leading cause of death in the non-coronary intensive care units. It kills more than 200,000 patients in the US alone each year [1]. Globally, 20–30 million patients are diagnosed and over 8 million lives are lost annually with sepsis and septic shock [2]. The immune response transitions from an early/hyper-inflammatory to a late/hypo-inflammatory phase during sepsis progression [3,4]. Although significant (1/3rd) sepsis-related mortality occurs during the hyper-inflammatory phase of sepsis, a majority of the sepsis-related deaths occur during the hypo-inflammatory/immunosuppressive phase, which is characterized by endotoxin tolerance [4,5,6]. Sepsis hyper-inflammation is associated with immune cell activation to enhance phagocytosis/ pathogen killing and is characterized by increased pro-inflammatory cytokines/chemokine expression [7]. Hyper-inflammation is a pro-oxidant state with increased expression of reactive oxygen species (ROS) and reactive nitrogen species (RNS), etc. [8]. Hypo-inflammatory phase is cytoprotective towards the immune cells with decreased pro-inflammatory (vs. hyper-inflammation) and increased anti-inflammatory cytokine expression [9,10]. Importantly, the hypo-inflammation is associated with impaired phagocytosis/bacterial killing, leaving the host susceptible to secondary infections. Multiple organ dysfunction with impaired mitochondrial function during the hypo-inflammatory phase is responsible for the increased mortality of sepsis [6,11,12].

During the hyper-inflammatory phase, a pro-oxidant generation helps the immune cells with bactericidal activity against invading pathogens [13]. Superoxide ions are produced during the normal consequence of energy production by mitochondria [14]. To balance these oxidants, cellular and mitochondrial antioxidants are activated [15]. Under physiological conditions, the production of pro- and anti-oxidant pathways is tightly controlled. However, the balance between pro- and anti-oxidants is dysregulated during sepsis and septic shock [16]. Compromised anti-oxidant capacity in patients with sepsis with overwhelming ROS/RNS generation leads to oxidative/nitrosative stress [13,17,18]. The overproduction of ROS dysregulates cellular structure/functions. Specifically, the mitochondrial dysfunction ultimately leads to tissue hypoxia and organ dysfunction [19,20]. Additionally, damaged mitochondria (mt) release a plethora of molecules, including mtDNA, ATP, cardiolipin and cytochrome c, triggering a pro-inflammatory response [21].

Epigenetic changes, defined as an impact of environmental modifications on gene expression without a change in gene sequence [22], include DNA methylation, Histone modifications and regulation by non-coding RNAs [23,24,25]. DNA methylation results in either gene activation or repression, depending upon the location of the gene and regulatory components [26,27]. Preclinical studies show that the inhibition of DNA methylation decreases hyper-inflammation and organ failure [11,28,29]. Histone modifications are implicated in converting active euchromatin (unwound chromatin) into reversibly silent heterochromatin (tightly wound chromatin), thus masking the genes from transcription factors to silence them [23,30]. Similarly, non-coding RNAs regulate gene expression both transcriptionally and post transcriptionally [31]. Thus, complex epigenetic modifications are critically important in driving gene transcription during sepsis [32].

Sirtuins (SIRTs) are a highly conserved family of NAD+ (Nicotinamide adenine dinucleotide)-sensor proteins. With a common function as deacetylase enzymes, the seven members (SIRTs 1-7) have several Histone and non-Histone targets [33]. The SIRTs share a conserved 275-amino-acid catalytic core domain and are dispersed throughout cell compartments: nuclear (SIRT1, SIRT6, and SIRT7); cytoplasmic (SIRT2); and mitochondrial (SIRT3, SIRT4, and SIRT5) [34,35,36]. Sirtuins regulate several cellular processes associated with oxidative stress signaling [35]. SIRT1, SIRT3, and SIRT5 protect cells from ROS/RNS damage, whereas SIRT2, SIRT6, and SIRT7 modulate key oxidative stress genes and their mechanisms [35]. Sirtuins are important epigenetic regulators via the deacetylation of Histones themselves and/or interactions with other proteins controlling Histone acetylation or DNA methylation enzymes [37,38]. The main focus of this review is to discuss the role of sirtuins in regulating oxidative stress and epigenetic modifications during sepsis and septic shock.

## 2. Sirtuins and Redox Regulation of Sepsis

Oxidative stress is integral to any inflammatory response, including sepsis. Mitochondria are not only a hub of energy and pro-oxidant molecule generation but are also vulnerable to damage by the same pro-oxidants. The literature overwhelmingly suggests that the ROS/RNS act as signaling molecules during sepsis [17]. Sirtuins, while integral to the oxidative stress response, are also prone to direct oxidation themselves, adding further complexity to the role of sirtuins. The following sections describe the interactions between sepsis-oxidative stress and sirtuins.

### 2.1. Mitochondrial Redox and Sepsis

The multiple organ failure, responsible for increased mortality in sepsis, is associated with cell death/dysfunction rather than the structural damage to the tissue itself [39]. Tissue hypoxia is crucial for organ dysfunction [40]. Sepsis non-survivors show impaired tissue oxygen consumption despite adequate oxygen supply [41,42]. Mitochondrial dysfunction is implicated in tissue hypoxia and, ultimately, organ failure in sepsis [11,43].

The energy/ATP production occurs at the inner membrane of mitochondria by electron transport chain (ETC)/oxidative phosphorylation [43]. ROS byproducts along with superoxide and hydrogen peroxide (H_2_O_2_) are generated as a part of this process [43]. Superoxide and H_2_O_2_ are highly reactive and short-lived but damage the surrounding molecules if not controlled [43]. Mitochondrial antioxidant systems neutralize these ROS under normal physiological conditions [43]. Mitochondria themselves are the targets of ROS [14,44]. In addition to ROS, the mitochondrial ETC also produces reactive nitrogen species (RNS) including nitric oxide with an unpaired electron and peroxynitrite [43]. In addition, the nitric oxide is produced by de novo synthesis by the inducible nitric oxide synthase (iNOS) during sepsis [43]. In general, super oxide dismutase (SOD) quickly converts superoxide anion to hydrogen peroxide and water. However, the interaction between superoxide anion and nitric oxide to form peroxynitrite is even more rapid and takes place both in the cytoplasm and mitochondria [17].

Peroxynitrite causes severe mitochondrial damage during sepsis [17]. Poly (ADP ribose) polymerase (PARP) is activated in response to single stranded DNA breakage in the nucleus by peroxynitrite [17]. PARP catalyzes the cleavage of NAD+, resulting in decreased NAD+ levels and an increased NADH/NAD+ ratio [45,46]. A high NADH/NAD+ ratio further increases superoxide production in the mitochondrial complex I [14] and raises the H_2_O_2_ levels. Excessive H_2_O_2_ leaks out of the mitochondria to promote NF-κB activation [17]. The pharmacological inhibition of PARP protects the mitochondria from peroxynitrite damage and decreases sepsis-induced mitochondrial impairment [46,47].

Defective anti-oxidant and/or overwhelming pro-oxidant production leads to oxidative stress, which, in turn, leads to a significant damage to lipids, proteins, and nucleic acids in the cytoplasm and mitochondria [43]. Mitochondrial DNA (mtDNA), due to its vicinity to the ETC, is particularly prone to oxidative damage [48]. Damage to mtDNA further leads to a series of ROS production and further mtDNA damage, setting up a vicious cycle [43]. In addition, ROS generation leads to the peroxidation of cardiolipin, a mitochondrial inner membrane lipid vital for energy metabolism [49,50]. These events result in an alteration in the mitochondrial inner membrane permeability, pore opening, organelle swelling, and cytochrome c release, ultimately affecting ATP generation with continued ROS production [51,52].

The damaged mitochondria are cleared through mitophagy/autophagy [53]. However, the recovery processes such as mitochondrial biogenesis to manage the depleted mitochondrial numbers are impaired during sepsis [12,54]. Anti-oxidant therapies are reported to inhibit mitochondrial swelling and cytochrome c release, indicating an important role of ROS associated mitochondrial dysfunction [55].

#### Sirtuins and Mitochondrial Redox

Sirtuins are known anti-oxidants [56,57]. The nuclear SIRT1 and mitochondrial SIRT3 regulate mitochondrial biogenesis during sepsis [58,59]. SIRT1 activates peroxisome proliferator-activated receptor γ coactivator-1alpha (PGC1α) which is essential for mitochondrial biogenesis and oxidative phosphorylation [60,61]. PGC1α stimulates several transcription factors, such as *nuclear factor-erythroid-derived 2-like* (NRF) 1 and 2, Estrogen-related receptor alpha (ERRα), Peroxisome proliferator-activated receptor alpha (PPARα), and Mitochondrial transcription factor A (TFAM), increasing the transcription of mitochondrial proteins and enzymes [62]. PGC1α expression is reported to increase during hypoxia and ROS production [63].

Mitochondrial SIRT3 regulates mitochondrial metabolism and oxidative stress [64,65,66,67]. Several enzymes involved in Kreb’s cycle and oxidative phosphorylation are hyper-acetylated in SIRT3 knock out mice, indicating a direct deacetylating role of SIRT3 [64,68,69]. Furthermore, SIRT3 deficient mice show reduced complex I function in the ETC followed by oxidative damage, decreased tissue ATP, and a significant increase in pro-inflammatory cytokine levels [64]. On the other hand, SIRT3 overexpression preserves mitochondrial function and decreases oxidative damage [64]. SIRT3 regulates oxidative stress by modulating both the formation and neutralization of ROS [70]. Specifically, SIRT3 directly activates manganese superoxide dismutase 2 (SOD2), a superoxide scavenger, increases reduced-glutathione levels by deacetylation, and activates mitochondrial isocitrate dehydrogenase 2 (IDH2), leading to increased Nicotinamide adenine dinucleotide (NADPH) levels [71,72]. During septic shock, the ROS cause mitochondrial membrane depolarization and activation of the membrane permeability transition pore (mPTP) [40,73,74]. SIRT3 is reported to deacetylate and stabilize cyclophilin D, an important element of mPTP [75]. In SIRT3 knockout mice, calcium challenge mediated mitochondrial enlargement is reversed by cyclosporine A, an mPTP inhibitor [75].

Although a limited number of studies show the specific role of SIRT3 in sepsis, SIRT3 plays an important role in mitigating inflammation in general, oxidative damage, and organ dysfunction [76]. The protective role of SIRT3 against mitochondrial damage in acute kidney injury (AKI) with the murine sepsis model is reported [77]. Similarly, SIRT5 activates SOD1, IDH1, and IDH2 enzymes to detoxify ROS [74,78]. Either SIRT3 or SIRT5 gene-deletion mice are prone to age-associated diseases [79,80]; however, conflicting results are also shown in breast cancer, indicating that silencing SIRT3 sensitizes the cancer cells for cytotoxic treatments [81]. SIRT5 reverses endotoxin tolerance in the macrophages by supporting the acetylation of NF-κB p65 [82]. Independent of its deacetylase activity, SIRT5 competes with SIRT2, which inhibits the deacetylation of NFκB p65 and leads to NFκB p65 acetylation (activation) and increased pro-inflammatory response [82]. However, the exact role of SIRT5 in sepsis and its therapeutic potential need to be fully elucidated [82,83]. SIRT4 increases late in sepsis in the monocyte mitochondria by an unknown feedback mechanism to the nucleus. SIRT4 represses Pyruvate dehydrogenase kinase 1 (PDK1) and stimulates pyruvate dehydrogenase complex (PDC) decarboxylation to acetyl CoA, which increases both glycolysis and the mitochondrial energy index in monocytes and breaks immune tolerance [84,85].

### 2.2. Redox Signaling in Sepsis

During hyper-inflammation, excessive production of ROS [Superoxide (O_2_^−^), Hydroxyl radical (OH), Hydroperoxyl radical (OOH), Peroxyl radical (ROO)], RNS [Nitric oxide (NO), and Nitrogen dioxide (NO_2_) radical] occur in the circulating immune cells and in the affected organs [40,86]. These ROS and RNS act as a first line of defense against the invading pathogen. Intracellular superoxide levels increase through NADPH oxidase, Cyclooxygenase-2 (COX-2), xanthine oxidase, and ETC in the mitochondria during sepsis [87]. Superoxides are neutralized by SOD under physiological conditions, but not during sepsis [88,89]. In addition, the entry of ROS directly from the plasma (extracellular) into the cell also stimulates NADPH oxidase and COX-2 expression [18,90,91]. Microbial products activate NADPH oxidase-2 (NOX-2) via Toll like receptors (TLR) mediated pathway [92]. During sepsis, the initial surge in superoxide production in endothelial cells is eradicated by NADPH oxidase inhibitors [87]. Evidence suggests that the inhibition of NOX-2 prevents sepsis induced cardiomyopathy in mouse models [93]. Similarly, COX-2 inhibition leads to a decreased production of peroxynitrite in experimental models of sepsis [90,94]. These data indicate the importance of limiting excessive ROS generation to prevent sepsis-related hyper-inflammation; however, the clinical value of this strategy is questionable due to limited opportunities to truly prevent sepsis in the clinical world.

#### 2.2.1. Redox Signaling Pathways in Sepsis

**Figure 1:**
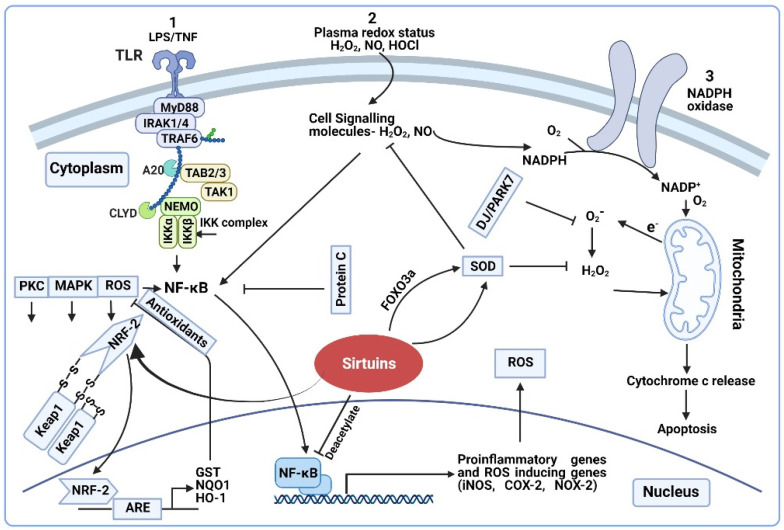
Regulation of ROS/RNS on transcription sensitive genes. (1) Activation of the TLR4 pathway by LPS/TNF-α and stimulation of NF-κB mediated proinflammatory cytokines and oxidative stress generation, further activation of NRF2 by ROS. (2) Plasma redox status as intracellular signaling molecule and activation of NF-κB. (3) Plasma ROS induce cellular expression of NADPH oxidase and production of superoxide. Oxidative stress leads to mitochondrial dysfunction, cytochrome c release and apoptosis. Schematic representation shows sirtuins’ interaction with NRF-2 (which regulates the expression of antioxidant and detoxification genes) and SOD (which either activates SOD directly or through the FOXO3a transcription factor); Sirtuins block the NF-κB mediated transcription of several proinflammatory genes through the deacetylate NFκB p65 subunit.

The altered redox status of a septic patient’s plasma affect the intracellular pro-oxidant levels since some of these oxidants, such as H_2_O_2_, NO, and Hypochlorous acid (HOCl) cross the cellular membrane [8,95]. After entering the cell, H_2_O_2_ and NO trigger a cascade of secondary reactions to produce more ROS. Highly reactive HOCl has a limited diffusion rate in various biological systems, hence, only a small fraction of HOCl enters the cell, where it leads to protein, DNA, lipid, and mitochondrial damage [96,97]. H_2_O_2_ and NO act as signaling molecules activating a specific set of genes through a number of transcription factors at the nuclear level such as the Activator protein-1 (AP-1), NRF-2, Cyclic adenosine monophosphate (cAMP) response element binding protein (CREB), Heat shock factor 1 (HSF1), Hypoxia-Inducible factor 1(HIF-1), Tumor Protein 53 (TP53), and NF-κB [98,99]. Among them, the NF-κB plays a central role in sepsis [18] (Figure 1). We describe the effect of the genes involved in various major pathways below.

*NF-kB pathway*: Upon entry from plasma, the H_2_O_2_ causes a cascade of intracellular events resulting in the release of NF-κB from its inhibitor IκB [100]. The reduced state of the dynein light chain protein LC8 controls the NF-kB [101]. Specifically, H_2_O_2_ induces the dimerization of LC8 via a disulfide bond formation due to oxidation, promoting the phosphorylation and activation of NF-kB through unmasking IκB [101]. Activation of NF-kB occurs through these and several other pathways, including tumor necrosis factor (TNF-α) [18]. NF-κB activity is known to be significantly higher in sepsis-non-survivors vs. survivors [102,103]. The binding of NF-κB to the DNA initiates several redox-active enzymes gene expression including iNOS, COX-2, and the generation of NO and O_2_^−^ as by-products [90,104]. iNOS expression is also regulated by hypoxia inducible factor-1, which is under the control of ROS [105]. During early sepsis, increased iNOS expression is observed in almost all vital organs [45,106,107]. Increased NO production and elevated nitrite/nitrate levels positively correlate with sepsis-severity [108].

*Nuclear factor erythroid-derived 2-like 2 (NRF-2) pathway*: NRF-2, is an important regulator of oxidative stress in sepsis [109]. NRF-2 regulates the antioxidant response element (ARE)-mediated transcription of multiple anti-oxidants that neutralize the harmful effects of ROS [110,111,112]. In general, NRF-2 interacts with Kelch-like ECH-associated protein 1 (KEAP1). ROS oxidize the redox sensitive cysteine residues on KEAP1, resulting in the dissociation of KEAP1 from NRF-2 in the cytosol [17]. With a scaffolding protein that binds NRF-2 and Cul3 ubiquitin ligase for proteasome degradation, NRF-2 translocates to the nucleus, heterodimerizes with small musculoaponeurotic fibrosarcoma (sMAF) protein, and binds to AREs for the downstream expression of antioxidant genes [113]. NRF-2 deficient mice exhibit increased mortality, while transgenic mice with NRF-2 stabilization show decreased mortality with polymicrobial sepsis [114,115]. Other than ROS, signaling pathways such as Extracellular signal-regulated kinase (ERK), Mitogen activated protein kinase (MAPK), and Phosphoinositide 3-kinase (PI3K) also activate NRF-2 [116]. NRF-2-regulation by pro-inflammatory cytokines is reported as well [109].

#### 2.2.2. Sirtuins and Redox Signaling

Metabolic processes utilize reduced compounds such as glucose and fatty acids, which, upon oxidation, release energy transmitted via NAD+ reduction to NADH [35]. Sirtuins are NAD+ dependent deacetylase enzymes, where the coenzyme NAD+ is involved in the reduction and oxidation (NADH/ NAD+) reactions. The NAD+ dependency of sirtuins, suggest their importance as regulators of cellular homeostasis. Sirtuins are the ideal enzymes to regulate redox reactions by modulating transcription factors controlling antioxidant levels and cellular NAD+/NADH ratios [117]. The NAD+/ NADH ratio is crucial for maintaining cellular redox homeostasis and metabolism [118]. The oxidative stress also modulates sirtuins function by the direct oxidation of redox sensitive cysteines [119,120].

NFĸB is one of the direct and downstream targets of both SIRT1 and SIRT2, and both modulate the NFκB activity [121,122]. SIRT1 deacetylates the RelA (p65) subunit of NFĸB to block the transcription of several pro-inflammatory genes, including interleukin-6 (IL-6), TNF-α, cytokine-induced neutrophil chemoattractant (CINC), COX2, and Intercellular adhesion molecule-*1* (ICAM-1) [121,123]. SIRT1 deletion leads to elevated cytokine and ICAM 1-expression with lipopolysaccharide (LPS) challenge via increased NFĸB activation [124]. We showed both SIRT1 and SIRT2 deacetylate NFκB p65 subunit in sepsis [125,126]. Evidence suggests that cyclin B/CDK-1 modulates SIRT1 phosphorylation and activity [127], while oxidative stress regulates B/cyclin-dependent kinase 1(CDK1) [128]. SIRT1 activity is modulated by s-glutathionylation. S-glutathionylation inactivates SIRT1 and SIRT1-p53 mediated apoptosis [129]. Similarly, glutathione adducts regulate SIRT1 activity and in turn regulate SIRT1 function in metabolic syndrome and liver disease [130]. Furthermore, SIRT1 is also regulated by nitrosylation [131] and Redox Factor-1 (APE/Ref-1) [132], suggesting a two-way relationship between oxidative stress and SIRT activity; oxidative stress regulates SIRT1, while SIRT1, in turn, regulates oxidative stress. Oxidative stress regulates sirtuin, specifically SIRT1 activity, by changing its binding to regulatory proteins [133]. Deleted in breast cancer 1 (DBC1) [134] and an active regulator of SIRT1 (AROS) [135], the two main protein regulators of SIRT1 are controlled by oxidative stress [135]. Oxidative stress increases the interaction of SIRT1 with its protein inhibitor, DBC1, through the phosphorylation of DBC1 (Thr454), which leads to SIRT1 inhibition [136]. Similarly, AROS deficiency decreases the SIRT1 mediated response to oxidative stress in cells.

NRF-2 is regulated by SIRTs as well. SIRT2 changes the total and nuclear NRF-2 levels by deacetylating the NRF-2 via AKT phosphorylation, suggesting that SIRT2 is an important regulator of AREs [137,138]. SIRT1 regulates NRF-2; SIRT1-knockdown inhibits the expression of NRF-2-mediated antioxidant enzymes [139]. During oxidative stress, SIRT6 co-activates NRF-2 in human mesenchymal stem cells (hMSCs) [140]. During oxidative stress, SIRT1 and SIRT2 deacetylate the forkhead box transcription factor 3a (FOXO3a), which regulates a wide variety of antioxidant defense genes [141,142,143].

Our earlier studies implicate an obesity-specific role of SIRT2 in innate immune cells during sepsis [126]. In obese-sepsis mice, the exaggerated hyper-inflammation is associated with decreased SIRT2 levels, whereas prolonged hypo-inflammation is associated with a sustained increase in SIRT2 expression [126]. The oxidative stress of obesity with sepsis directly oxidizes SIRT2 to deactivate its deacetylation-function and exaggerates hyper-inflammation. Decreased levels of oxidized-SIRT2 with increased total-SIRT2 levels occur during prolonged hypo-inflammation [119]. Moreover, SIRT2 inhibition during hypo-inflammation improves survival in *ob/ob* mice [126].

Evidence suggests that the SIRT6 oxidation during the hyper-inflammation of sepsis modulates its glycolytic function [120]. The redox-dependent s-sulphenylation of cysteine 18 on SIRT6 modulates binding and its suppressive activity on HIF-1α [144], suggesting the redox regulation of SIRT6 activity.

SIRT6 overexpression inhibits oxidative stress and glioblastoma cell growth by inhibiting oxidative stress via suppressing malondialdehyde (MDA) level, and increasing CuZu/Mn-SOD activity [145]. SIRT6 mono-ADP ribosylation of BRG/BRM-associated factor (BAF) promotes its recruitment on chromatin remodeling complex and enhance the activation of NRF-2 target genes during oxidative stress [146]. SIRT6 deficient cells are sensitive to oxidative stress and show reduced capacity for DNA repair whereas SIRT6 knockout mice display many symptoms of premature aging [147]. Although known in cancer and ageing literature, the role of SIRT6 in modulating sepsis-induced oxidative stress via SOD or activation of NRF-2 target gene expression needs further evaluation.

## 3. Sirtuins and Epigenetic Regulation of Sepsis

### 3.1. Epigenetic Regulation

**Figure 2:**
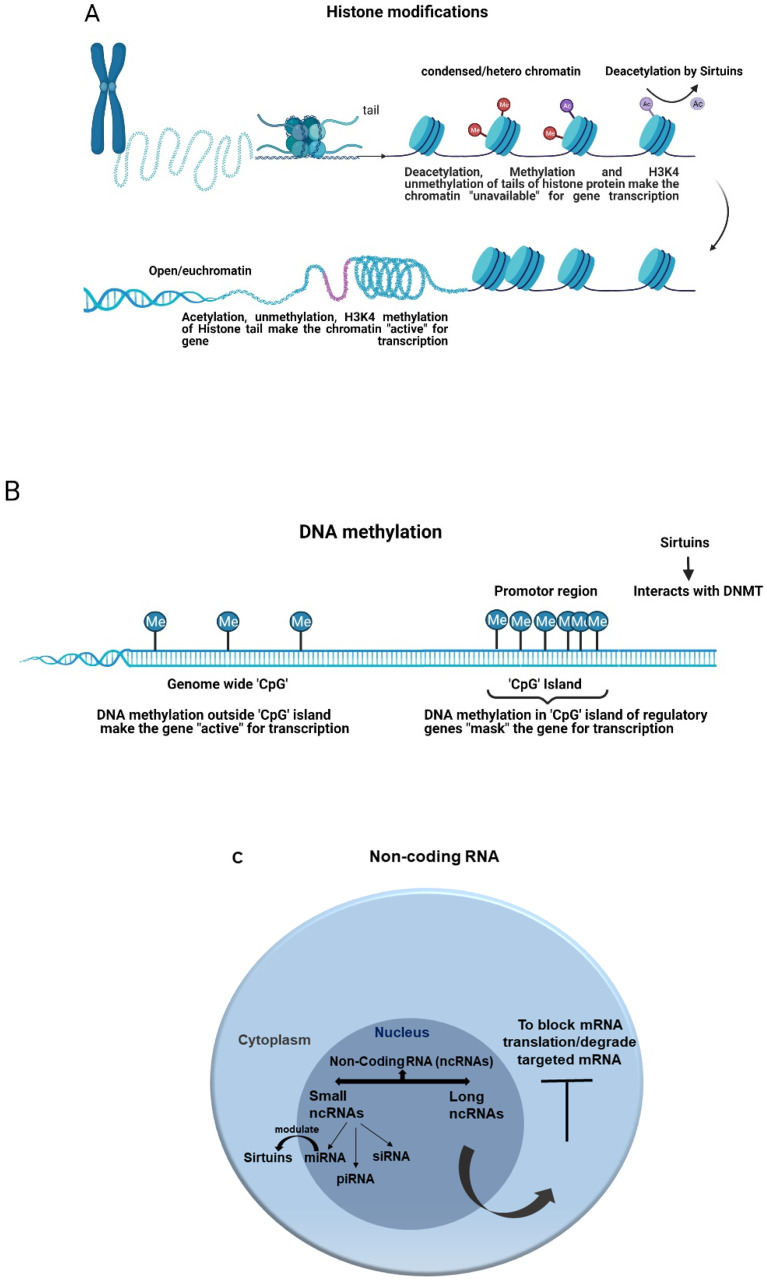
General epigenetic modifications and the role of sirtuins. (**A**) Histone modification and deacetylation by sirtuins. (**B**) DNA methylation and sirtuins interaction with DNA methyl transferase. (**C**) Non-coding RNA and sirtuins modulation by miRNA.

Epigenetics is a regulatory mechanism for gene expression produced by chemical modifications either in DNA or Histones and transcription regulation through non-coding RNAs without any changes in the original gene sequence [25]. All of these alterations reshape the chromatin to promote or hamper gene expression. The three major mechanisms of epigenetic regulation during sepsis are described below (Figure 2).

*DNA methylation*: DNA methylation is the most extensively studied epigenetic modification. In general, DNA methylation marks the gene being silent by masking it from gene expression. During this process, a methyl group (CH3) is added by DNA methyltransferase (DNMT) enzymes to 5′ carbon of cytosine, located in 5′-Cytosine-phosphate-Guanine-3′ (CpG) dinucleotide regions and demethylated by the ten-eleven translocation (TET) enzymes [133]. Although CpG dinucleotides are present genome-wide, they are asymmetrically located in promotor regions, called CpG Island (CGI) [148,149]. The addition or removal of methyl group in the DNA alters the chromatin structure and protein binding, ultimately modifying the gene expression. Hyper-methylation in CpG Island of the promoter region is commonly linked with gene *repression*, whereas hypo-methylation is associated with gene *activation* [150]. In contrast, DNA methylation outside CpG Island is associated with gene activation [151]. This relationship is not constant, making it difficult to understand the impact of DNA methylation on individual genes [26,152].

*Histone modifications*: Histones are a family of small, positively charged proteins, consisting of H2A, H2B, H3, and H4 as core proteins, where the negatively charged DNA double helix is wrapped around them to form a structure called nucleosome, a part of the chromatin [25,153]. Like other cellular proteins, Histones are prone to various posttranslational modifications such as acetylation, methylation, phosphorylation, ribosylation, ubiquitination, sumoylation, and glycosylation that play major roles in DNA accessibility [154,155]. In general, phosphorylation and ribosylation support euchromatin (unwound chromatin) formation to make a gene accessible to transcription [156,157,158,159]. Sumoylation triggers gene silencing through heterochromatin formation [160,161]. Ubiquitination has dual roles [162,163]. The acetylation and methylation of Histone are catalyzed by Histone Acetyl Transferases (HAT) and Histone methyl transferase (HMTs) enzymes at specific residues, respectively [164]. Some Histone modifications such as H3K4me3 (trimethylation of lysine 4 of Histone H3) and H3K27ac (acetylation of lysine 27 of Histone H3) consistently lead to the active transcription of the gene target [154]. Likewise, the methylation of Histone H3 at lysine 27 (H3K27me) results in gene suppression [165]. The crosstalk between DNA methylation and Histone modifications leads to further chromatin modifications that eventually promote an active or inactive conformation of chromatin [155].

*Non-coding RNAs*: Non-coding RNAs (ncRNAs) regulate the expression of other genes post-transcriptionally [31]. Transcriptional ncRNAs are classified into small ncRNAs and long ncRNAs (lncRNAs). Small ncRNAs are further divided into microRNAs (miRNAs), PIWI (P-element Induced Wimpy)-interfering RNAs (piRNAs), and small interfering RNAs (siRNAs) [151]. Among them, the Small ncRNAs are most widely studied. Especially, miRNAs post transcriptionally silence almost 60% of protein-coding genes [166]. The miRNAs are formed inside the nucleus and are released into the cytoplasm to post-transcriptionally interfere or degrade their target messenger RNAs (mRNAs). We describe the role of epigenetics in the pathophysiology of sepsis and the epigenetic regulation of sepsis by SIRTs below.

### 3.2. Epigenetics Regulation of Sepsis

During the early/acute phase of sepsis and the stimulation through pattern recognition receptor (PRR) signals, hundreds of pro- and anti-inflammatory genes are transcribed. A study of human endotoxemia revealed the expression of over 3700 genes changed within 2 h of exposure due to the alteration in DNA methylation and Histone modifications, including H3K27 acetylation and H3K4 methylation [167]. These epigenetic modifications activate genes involved in cytokine and interferon signaling [168].

Histone acetylation is important in the regulation of pro-inflammatory genes such as TNF-α, IL-6, and IL-1β [169,170]. Five families of HATs enzymes that add acetyl group are identified so far. These enzymes use acetyl-CoA as a substrate and target lysine residues on H3 and H4 [171]. Similarly, in humans, eighteen Histone deacetylases (HDACs) that remove acetyl groups are found and classified into four groups based on their sequence-similarity. Class I, II, and IV HDACs are known as “classical” Zn^2+^-dependent HDACs and are widely studied. Class III HDACs, also known as sirtuins, require NAD^+^ as a substrate for lysine deacetylation [172]. HATs and HDACs have multiple non-Histone targets, crucial for various cellular processes such as metabolism and cell cycle [172].

An association between DNA methylation and pro-inflammatory response is reported. For example, H3K4me3 controls the repositioning of two nucleosomes on the NF-κB binding of the TNF-α promoter, exposing the binding site and ultimately leading to the up-regulation of TNF-α transcription in LPS-responsive cells [173,174]. A transcriptomic study found a crucial role for epigenetics during the acute inflammation of sepsis; specifically, several epigenetic-modifying enzymes such as DNA methylation and Histone-modifying proteins were differentially expressed during early sepsis [175].

Epigenetic modifying drugs inhibit the acute inflammatory response of endotoxemia in animal models through epigenetic mechanisms [25]. Decitabine, a chemotherapy agent and DNA methyl transferase inhibitor (DNMTi), is reported to reduce macrophage activation, migration, and adhesion during endotoxin exposure by downregulating the key inflammatory genes, iNOS, and chemokines in a mouse model [176]. Procainamide, an antiarrhythmic agent and a DNMTi, is reported to decrease hypotension and hypoglycemia and improve survival in a rat model of endotoxemia [29]. DNMTi treatment was given either prior to or along with the LPS exposure in these studies, indicating a “preventive” role for epigenetic inhibition; however, the clinical implications of this strategy remain unclear.

Non-coding (ncRNAs) play an important role in the pathogenesis of sepsis [177]. Analysis of the co-expression network of protein-coding and long ncRNAs (lncRNAs) in septic and healthy neutrophils indicates that some of the lncRNAs alter gene expression, regulate the translation of proteins, and are involved in regulatory loops during sepsis [178]. A transcriptome study in the blood leukocytes of septic patients and healthy individuals revealed that both lncRNAs and small ncRNAs (to a lesser extent than the lncRNAs) encounter significant changes in septic patients [179]. Thus, ncRNAs in general can serve as diagnostic markers for sepsis and therapeutic targets.

Hypo-inflammation is characterized by “endotoxin tolerance,” where the immune cells are unable to respond secondary inflammatory stimuli [180,181]. Epigenetic modifications play an important role in endotoxin tolerance [173]. During progression towards endotoxin tolerance in an immune cell, the Histone methyltransferase G9a is recruited to the TNF-α promoter, resulting in the dimethylation of Histone 3 lysine 9 (H3K9) and the recruitment of DNMTs to further methylate the TNF-α promoter, leading to endotoxin-tolerance [182]. Pro-inflammatory cytokine IL-1β is downregulated by similar epigenetic regulation during sepsis [183].

miRNA-mediated epigenetic modifications play a major role in endotoxin tolerance as well. Several miRNAs are known to downregulate TNF-α expression during the endotoxin-tolerant phase of monocytes [25]. Among them, miR-221, miR-579, miR125b, and miR146a are induced after endotoxin exposure through TLR4 signaling [184]. Specifically, miR-221, miR-579, and miR125b bind directly to 3′ untranslated mRNA regions of TNF-α to block TNF-α translation [184]. Interestingly, miR-146a regulates the binding of repressor RelB to the NFκB binding site of TNF-α promoter, leading to silencing of the gene. Subsequently, miR-146a also regulates the pathway that supports an assembly of the translational repressor complex that further prevents TNF-α gene expression [185].

Sirtuins and epigenetic regulation of sepsis: Prolonged endotoxin tolerance leads to a metabolic transition from high-energy glycolysis to low-energy lipolysis [186]. Epigenetics play an important role in regulating this transition. The aforementioned nutrient sensor, NAD+ dependent SIRT1, a member of the type III HDAC enzyme family, acts as a metabolic sensor of the cell. During the transition from hyper- to hypo-inflammation, an increase in nuclear NAD+ activates SIRT1, which supports the regulation of the key inflammatory genes such as TNF-α and IL-1β through NFκB p65 deacetylation, indicating the central role played by SIRT1 in the regulation of immune-suppression during sepsis [36,187,188]. Recent reports from our group showed that EX-527, a SIRT1 inhibitor, reverses hypo-inflammation in sepsis mice and improves survival [3,189].

We, along with others, have showed that, during sepsis, monocyte transition from the hyper- to hypo-inflammatory phase involves gene specific switching of euchromatin (hyper-inflammation) to heterochromatin (hypo-inflammation) [30,190]. Mechanistically, we observed that this transition requires a switch from NFκB p65-activation to RelB-driven signaling through SIRT1 [3,191,192,193]. SIRT1 assists RelB binding on DNA, along with the alteration of Histones in the proximal promotors of pro-inflammatory genes, and reprograms DNA epigenetic code to form heterochromatin [10]. We showed that SIRT1 activates RelB during hypo-inflammation to orchestrate this epigenetic reprogramming [58]. Similarly, SIRT2 regulates epigenetic remodeling through the deacetylation of Histone H3K18, which leads to transcriptional repression during *L. monocytogenes* infection [194]. SIRT2 dephosphorylation at Serine 25 residue is necessary for SIRT2 chromatin association during *L. monocytogenes* infection [195]. Both SIRT1 and SIRT2 directly interact with the DNMT3B enzyme, which inhibits active Histone H3 modifications in pro-inflammatory genes such as TNF-α, IL1-β. Evidence suggests that SIRT1 and SIRT2 interact with the DNMT, specifically DNMT3A, during macrophage differentiation [38]. Interestingly, during inflammation, SIRT1 and SIRT2 inhibition prevents the hypermethylation of CpGs allowing pro-inflammation to occur, but does not actively participate in the hypomethylation of these CpGs, indicating a specific role for these sirtuins [38].

Micro RNAs modulate immune response via SIRT modifications. Reports indicate that SIRT1 is regulated by several mi-RNAs, including miR-34a, miR-181, and miR-217 [196,197]. The miR-138 exaggerates inflammatory responses in LPS induced animal and cell (macrophages) models by targeting SIRT1 and regulating the NF-κB and AKT Pathways [198]. Increased expression of miR-133a in sepsis patients and rodent sepsis aggravates an inflammatory response by targeting SIRT1 [199]. miR-199a is reported to increase in sepsis-induced acute respiratory distress syndrome (ARDS) [200]. In all of the above studies, the downregulation of miRNAs using their specific inhibitors upregulates SIRT1 and suppresss excessive inflammatory response both in vitro and in vivo sepsis models.

## 4. Conclusions

In conclusion, immune response in sepsis transitions from a hyper-inflammatory to a hypo-inflammatory phenotype. The oxidative stress during hyper-inflammation, while intended for pathogen clearance, starts a cascade of events leading to a transition to hypo-inflammation. The hypo-inflammation is cytoprotective towards the immune and other cells during sepsis. Oxidative stress and epigenetic modifications regulate the pro- and anti-inflammatory gene program during both the hyper- and hypo-inflammatory phases. SIRTs, the NAD+ sensor proteins, play an integral role in modulating oxidative stress and the epigenetic response and ultimately reprogram the immune response in sepsis. A phase-specific targeting of SIRTs can be a much-needed potential therapeutic strategy in sepsis.
